# Regulatory Multidimensionality of Gas Vesicle Biogenesis in *Halobacterium salinarum* NRC-1

**DOI:** 10.1155/2011/716456

**Published:** 2011-10-24

**Authors:** Andrew I. Yao, Marc T. Facciotti

**Affiliations:** Genome Center UC Davis, Department of Biomedical Engineering, University of California, Davis, One Shields Avenue, Davis, CA 95616, USA

## Abstract

It is becoming clear that the regulation of gas vesicle biogenesis in *Halobacterium salinarum* NRC-1 is multifaceted and appears to integrate environmental and metabolic cues at both the transcriptional and posttranscriptional levels. The mechanistic details underlying this process, however, remain unclear. In this manuscript, we quantify the contribution of light scattering made by both intracellular and released gas vesicles isolated from *Halobacterium salinarum* NRC-1, demonstrating that each form can lead to distinct features in growth curves determined by optical density measured at 600 nm (OD_600_). In the course of the study, we also demonstrate the sensitivity of gas vesicle accumulation in *Halobacterium salinarum* NRC-1 on small differences in growth conditions and reevaluate published works in the context of our results to present a hypothesis regarding the roles of the general transcription factor tbpD and the TCA cycle enzyme aconitase on the regulation of gas vesicle biogenesis.

## 1. Introduction

The halophilic archaeon *Halobacterium salinarum *NRC-1 regulates the production of gas vesicles in response to shifts in various environmental factors. While gas vesicles (protein complexes that sequester gasses likely through the hydrophobic exclusion of water) confer buoyancy, the functional relevance, long thought to be a means by which cells could escape anoxic subsurface environments for more oxygen-rich surface waters, has recently been brought into question [1]. In *Halobacterium salinarum *NRC-1, gas vesicle proteins are expressed from two gene clusters, *gvp1 *and *gvp2. *A copy of each gene cluster is found on the pNRC200 plasmid while pNRC100 encodes only the *gvp1 *cluster [2–4]. Both *gvp *gene clusters encode two divergent operons encoding *gvpACNO *and *gvpDEFGHIJKLM, *respectively [3]. Of the 14 genes in the gvp gene cluster, only 10 (*gvpACODEFGJLM) *have been characterized, and only a fraction is required for gas vesicle expression [4–6]. 

The number and relationships among environmental factors influencing gas vesicle biogenesis has revealed itself to be more complex than originally anticipated. Carbon sources and oxygen may both play a part in regulating the biosynthesis of gas vesicles at both the transcriptional and posttranscriptional levels. A number of studies have suggested that low dissolved oxygen content in the growth media can trigger gas vesicle biogenesis [7–10]. An extension of this hypothesis was used to explain the large increases in gas vesicle abundance observed in batch cultures entering the comparatively oxygen-depleted stationary phase of growth [11, 12]. Two recent studies, however, obfuscate this simple functional interpretation by demonstrating that arginine and citrate may also contribute to the regulation of gas vesicle biogenesis in *Halobacterium salinarum *strains PHH1, PHH4, and NRC-1 [1] and that glucose may also impact gas vesicle biogenesis in *Haloferax mediterranei *[13]. Hechler and Pfeifer [1] have also suggested that anaerobic conditions inhibit gas vesicle biogenesis. More recently, Kaur et al. have also shown increased gas vesicle accumulation in response to oxidative stress induced by H_2_O_2_ and paraquat treatment [14] signaling that perhaps other unknown factors may also contribute to the regulation of GV expression. In addition to the previously discussed set of environmental factors that influence gas vesicle production, it is curious to note that under some conditions GVs, typically cylindrical with conical caps, can vary greatly in size and shape depending on the environmental stimuli [15]. Therefore, the regulation of GV expression and assembly is multifaceted and can serve as an interesting model system for studying complex integration of environmental data into different physiological and morphological phenotypes.

**Figure 1:**
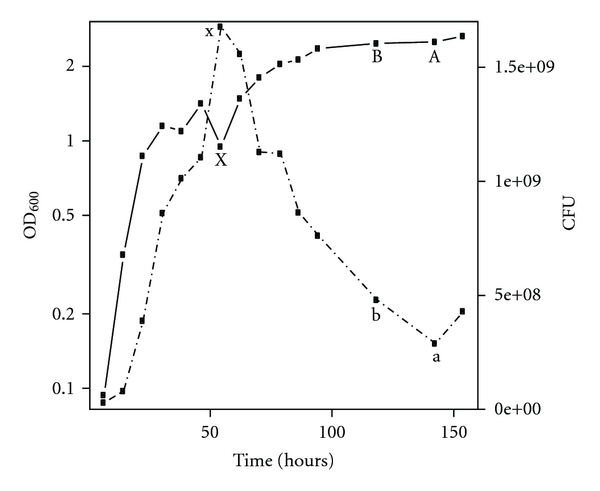
Growth of *Halobacterium salinarum *NRC-1. The *Halobacterium salinarum *NRC-1 growth curve derived from data originally presented in Facciotti et al. [12]. OD_600_, displayed against the left *y*-axis in logarithmic scale, is denoted by open circles. CFU, displayed against the right *y*-axis in linear scale, is denoted by closed circles. Points X and x (representing OD_600_ and CFU, respectively) mark the initial reference point for GV supplementary experiments. Point pairs A and a, and B and b, denote the corresponding OD_600_ and CFU data where the two largest CFU decreases were observed. Point pairs (A, a and B, b) along with reference points (X, x) were used to calculate the number of GVs released into media during stationary phase.

Two observations related to GV production during the growth of *Halobacterium salinarum *initially drew our attention to GV biogenesis. The first is the observation by Shand and Betlach [11] of an increase in optical density measured at 600 nm (OD_600_) that occurs in stationary phase cultures of *Halobacterium salinarum *NRC817 grown in complex media in brightly illuminated tissue culture flasks. The OD_600_ increased from OD_600_ ~1.0 to OD_600_ ~10.0 from the start of stationary phase to the final plateau. Counts of colony forming units (CFUs), however, show that the number of viable cells remains constant throughout this phase. The authors attributed the increase in OD_600_ to the accumulation of intracellular GVs, citing unpublished data [11]. More recently, Facciotti and coworkers [12] have noted a similar increase in OD_600_ during stationary phase of cells grown in ambiently illuminated, complex-media, shaken flask grown cultures of *Halobacterium salinarum *NRC-1 (Figure 1). In this report, OD_600_ increased from ~1.0 to ~2.5 from the start of stationary phase to the final plateau, a smaller increase in OD_600_ than that reported by Betlach and Shand [11]. By further contrast to Betlach and Shand [11], CFU counts decreased in the flask-grown cultures during stationary phase suggesting cell death and perhaps lysis. While increases in late stationary phase OD_600_ have been traditionally attributed to the accumulation of intracellular GV [16], the observation of an increase in OD_600_ in a stationary phase in which viability decreased was more mysterious. 

Several possibilities were suggested to account for the increase in OD_600_ in the latter flask-grown cultures, including light scattering from gas vesicles, cell clumping, and an increase in bacteriorhodopsin production, which is known to occur in stationary phase [12]. Two of these hypotheses were eliminated as cell clumping was not observed, and optical density readings measured at 700 nm (outside the main bR absorption peak) did not show any pronounced difference to those taken at 600 nm [12]. Therefore, the authors concluded that the most reasonable explanation of the available data was a release of gas vesicles from lysing cells [12]. Phase contrast images taken of the culture throughout its growth trajectory also showed abundant accumulation of small light-scattering bodies accumulating in late growth phases, reinforcing this hypothesis.

 This hypothesis was, however, challenged on the basis of insufficient physical evidence, despite historical evidence of strong light scattering by suspensions of purified gas vesicles from *Anabaena flos-aquae *[16, 17]. The core question, was whether or not free gas vesicles, in amounts expected to be released by lysing *Halobacterium salinarum *NRC-1 cells, could scatter sufficient light to account for the increase in overall OD_600_, despite decreased scattering from lysing cells. Given the recent interest surrounding GVs in halophilic archaea, we thought it would be worthwhile to formally address the hypothesis that GVs released from lysing *Halobacterium salinarum *NRC-1 cells can scatter light sufficiently to affect OD_600_ measurements.

 In this study, we demonstrate that free gas vesicles, in numbers proportional to what might be expected to be released by cell lysis in late phases of growth, scatter light sufficiently to account for the increase in optical density noted previously by Facciotti and colleagues [12]. We also demonstrate the influence of internal gas vesicles on optical density in *Halobacterium salinarum *NRC-1, confirming earlier observations of Walsby [17]. In the process, we have also observed that growth and GV production in *Halobacterium salinarum *is very sensitive to small changes in culture conditions. Together, our observations have also led us to reexamine previously published results and to propose a hypothesis regarding the roles of the general transcription factor TbpD and the TCA cycle enzyme aconitase in the regulation of gas vesicle biogenesis.

## 2. Materials and Methods

### 2.1. Culture Growth

*Halobacterium salinarum *NRC-1 and all mutants were grown in complex media (CM) consisting of 25% NaCl, 2% MgSO_4_·7H_2_O, 0.2% KCl, 0.3% Na-Citrate and 1% Oxoid^TM^ neutralized peptone (Fisher Scientific: New Hampshire, USA) [18]. Fifty milliliter cultures, grown in 125 mL unbaffled flasks (Corning, New York, USA) at 37°C, were inoculated with midlog preculture to an initial OD_600_~0.001 and shaken at either 100, 150, or 225 rpm, depending on the experiment. Colony forming units (CFU) were determined using the spread plate technique. Two different dilutions (5 × 10^7^ and 5 × 10^8^) were plated in triplicate for each time point. Optical density was measured using an Eppendorf Biophotometer (Hamburg, Germany). The program DataThief [19] was used to extract data from Shand and Betlach [11] for comparison and analysis.

### 2.2. Transmission Electron Microscopy (TEM)

*H. salinarum *NRC-1 was grown to stationary phase, OD_600_ = 1.7. Flasks were allowed to equilibrate on the lab bench for 24 h, and buoyant cells were harvested (see Figure S1 in Supplementary Material available online at doi:10.1155/2011/716456). Formvar-coated copper grids were immersed in the harvested cells for 1 min, and excess liquid was removed with filter paper. Two percent uranyl acetate, pH 4.0, was used to stain cells. Cells were examined with a Philips CM120 electron microscope (Amsterdam, Netherlands) running on 80 kV voltage and 11,000 X magnification. Images were taken using a Gatan MegaScan camera attached to the microscope. Gas vesicle dimensions were calculated with ImageJ software [20].

### 2.3. Isolation of Gas Vesicles

Gas vesicles were harvested using a modified version of the method described by Cohen-Bazier et al. [21] as described in [22]. Briefly, lawns of NRC-1 were grown on 2% CM agar plates at 37°C. 15 mL 1.0 mM MgSO_4_ with 10 *μ*L Benzoase (Novagen: Darmstadt, Germany) was poured onto the lawn and incubated at 37°C for 3 hours. Lysate was filtered through three sheets of Kimwipes, and filtrate NaCl concentration was adjusted to 10% (w/v). Gas vesicles were washed by overlaying of a 5% NaCl solution and centrifuged in a JOUAN CR3 centrifuge with a T40 Swing Bucket Rotor (Thermo Scientific, Mass, USA) at 60 xg overnight. Floating gas vesicles were harvested and rewashed three times with 5% NaCl.

### 2.4. Calculation of Gas Vesicle Scattering Factor (GVSF)

TEM images of vacuolated NRC-1 cells were taken as described above. GVs grown under conditions specified above were found to have a mean length of 350 nm and mean width of 200 nm (see Figure S2A), dimensions consistent with previous observations [5, 23]. To determine the GVSF, a typical gas vesicle was modeled as a rectangular solid (350 nm length, 200 nm width, and 200 nm height) whose surface is composed of cubic proteins. The cubic subunit dimensions were approximated using the molecular weight of GvpA, the main structural component of GVs, and the partial specific volume of porcine adenylate kinase [24], a protein whose external envelope can be approximated by a small cube. Since porcine adenylate kinase is larger than GvpA, 21 kDa versus 8 kDa, respectively, the edge dimensions of the cubic model for GvpA were scaled appropriately. A total of 43,810 proteins are required to cover the surface of a theoretical gas vesicle suggesting that the mass of each gas vesicle equals 1.14 × 10^−9^ *μ*g. 

The optical density at 600 nm was measured for serial dilutions of purified gas vesicles, dissolved in 5% NaCl. The serially diluted samples were then solubilized with SDS (final concentration 1.0%) and boiled for 10 mins at 95°C. Solubilized gas vesicle protein concentration was found using a BCA Assay Kit (Thermo Scientific, Mass, USA). Standards were prepared using diluted bovine serum albumin in a working range of 5–250 *μ*g/mL. Working reagent was prepared according to the protocol provided with the kit. 25 *μ*L of preprepared standard and sample dilutions were each pipetted into individual wells of a 96 clear flat bottom well plate (Corning: NY, USA). Two hundred microliters of working reagent were added to each well and mixed. The plate was placed at 37°C for 30 min then cooled to room temperature. Absorbance for each well was measured at 562 nm in an Infinite M200 Plate reader (Tecan: Männedorf, Switzerland). Protein concentration per OD_600_ unit was determined by dividing protein concentration by OD_600_ for the respective sample yielding a value of 44.1 *μ*g/mL/OD. Using the mass of individual gas vesicles calculated above, a GVSF was then determined to be 3.87 × 10^10^ GV/mL per OD_600_ = 1.0. We note that this specific GVSF is highly dependent on GV morphology (spherical v. spindle and size) and composition [16, 25] and applies to GVs isolated from *Halobacterium salinarum *NRC-1 grown in CM media into stationary phase cells. A similar calculation would need to be repeated for GVs grown in other conditions that show morphological differences from those studied herein. 

To verify that the analyzed proteins were predominantly gas vesicle proteins, this can lead into the 2.23 mg of purified gas vesicles were dissolved in 2% SDS and boiled for 10 minutes prior to loading on a 4–12% Tris-glycine gel (Invitrogen, Calif, USA). Gels were washed 3 times for 5 min with 200 mL of MiliQ water and then stained with 20 mL of Imperial Protein Stain (Thermo Scientific, Ill, USA) for 1 h. Gels were destained overnight with 0.5 M NaCl. Consistent with previous results [26, 27], stained gels showed much less protein material than expected indicating that gas vesicles were relatively pure. 

### 2.5. Measuring the Influence of Trace Elements on Growth Profile

An Infinite M200 Plate Shaker (Männedorf, Switzerland) was used to test how different concentrations of trace elements in CM affect the growth of *Halobacterium salinarum *NRC-1. Cells were diluted to an initial OD_600_ of ~0.01 in CM supplemented with either 0.25X, 0.5X, 0.75X, 1.0X, or 2.0X equivalent amounts of standard Fe^2+^ and Mn^2+^ concentrations of 18 *μ*M and 1.9 *μ*M, respectively. Two hundred microliters of diluted inoculum was loaded in replicates of 6 into the 60 innermost wells of a Nunc 96-well clear optical bottom plate (Thermo Scientific, USA). To prevent evaporation from the 60 experimental wells, the outermost ring of 36 wells were filled with 200 *μ*L of blank CM. The lid of the 96-well plate was sealed on with TempPlate Sealing Film (USA Scientific) and Scotch Tape (3M, Minn, USA). The plate was loaded into the Infinite M200 Plate Shaker set to shake orbitally with an amplitude of 2.5 mm at 37°C. Optical density for each well was measured at a wavelength of 600 nm every 30 minutes for 120 hours. OD_600_ values were converted to a path length of 1 cm to standardize readings taken with the Eppendorf Biophotometer (Hamburg, Germany).

### 2.6. Isolation and Growth of Gas Vesicle Minus (GV−) Mutants

Isolation of GV minus mutants was accomplished according to methods presented in Stoeckenius et al. [28] and DasSarma [29]. Briefly, *Halobacterium salinarum *NRC-1 cells were plated onto 2% CM agar plates. Once grown, purple translucent colonies (see Figure S2B) were selected and recultured in CM. The GV*−* strain was confirmed by inspection by phase contrast microscopy and by replating on 2% agar plates (see Figures S2B and S2C).

## 3. Results

### 3.1. Gas Vesicle Release from Lysed Cells Can Account for Increases in Culture OD_600_

We first tested the hypothesis proposed by Facciotti et al. [12] that light scattering from gas vesicles released from lysed stationary phase cells could account for the increase in OD_600_ reported in their manuscript (Figure 1). We did this in two ways. First, using the GVSF determined above, we calculated the theoretical increase in OD_600_ that might be expected from the number of cells Facciotti et al. [12] reported to have lost viability in stationary phase. The reduction of viable cells, assumed to derive completely from cell lysis, was determined by taking the difference in CFUs between point x during early stationary phase (maximum CFU, Figure 1, Point x) to point a during late stationary phase (minimum CFU) (Figure 1, Point a). Using a previously published count of GV per cell in *Halobacterium salinarum *NRC-1 (~80 GV/cell during stationary phase) [1], we calculated the expected number of gas vesicles released into the media from the lysed cells (1.39 × 10^9^ lysed cells). Using this previously published value was appropriate as both the intracellular occupation (viewed as bright refractive zones using phase contrast microscopy) and the average GV size (determined by electron microscopy) of our cells are very similar to what Hechler and Pfeifer report in their study [1]. The GVSF was then used to determine that 1.11 × 10^11^ gas vesicles should have been released and that these alone would account for an OD_600_ of 2.87. Adding the expected scattering (1.0  OD_600_ = 5 × 10^8^ cells/mL) for unlysed cells (OD_600_ ~0.6) results in an expected final OD_600_ of 3.47. This is nearly 1.0 OD_600_ unit larger than what was reported by Facciotti et al. [12] suggesting that released gas vesicles, alone, could indeed account for the observed increase in OD_600_.

The discrepancy in final OD_600_ derived from the calculation above and the data reported by Facciotti et al. [12] can be easily accounted for by two factors. First, it is possible that the total number of lysed cells is lower than what was assumed above. A decrease in CFU does not require all cells to lyse, and therefore, scattering from released GVs may be slightly lower than what we calculated above. Second, it is likely that the number of GVs expressed per cell is less than 80. While Hechler and Pfeifer [1] reported an average of ~80 GVs per cell, it is conceivable that the cultures grown by Facciotti et al. [12] contained fewer GVs per cell since cells begin to lyse at the onset of stationary phase when relatively fewer GVs have accumulated. In fact, reducing the average number of GVs per cell to 54 can bring the theoretical OD_600_ in agreement with the measured data.

**Figure 2:**
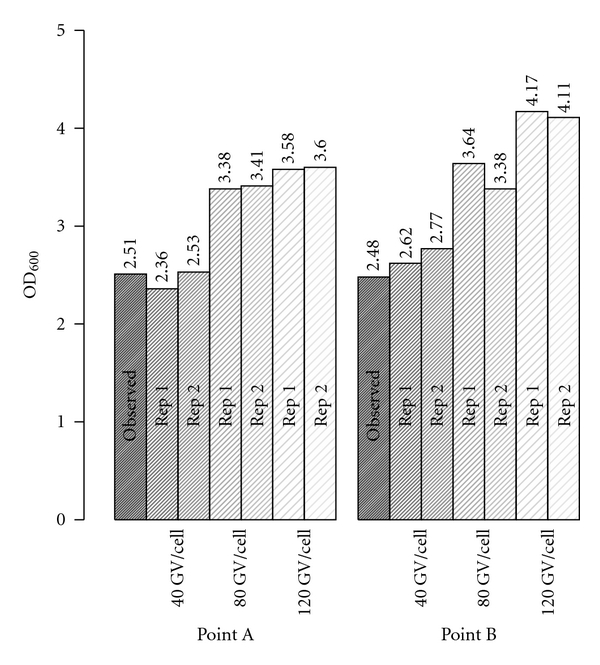
The contribution of free gas vesicles to light scattering at 600 nm. Gas vesicles were purified as described in Materials and Methods. To replicate the concentration of viable cells at points a and b (Figure 1) cells grown to OD_600_ = 0.4 were concentrated to OD_600_ = 1.0 and OD_600_ = 0.5, respectively. These OD_600_ represent a baseline of scattering form lightly-vacuolated/non-lysed cells at equivalent CFUs to points a and b in Figure 1. Purified gas vesicles were added to concentrated cells in amounts corresponding to the product of either 40, 80, 120 GV/cell and the total number of cells that were expected to have lysed between points x and a, and x and b. OD_600_ of the cell/GV mixtures was remeasured. Observed OD_600_ values from Figure 1 (points A and B), in addition to mixture OD_600_ are plotted as bar charts. OD_600_ values are included above each column.

Next, we empirically determined whether the addition of specific amounts of purified GVs to cell culture could account, in a predictable manner, for the increases in OD_600_ noted by Facciotti et al. [12]. In this experiment, purified *Halobacterium salinarum *NRC-1 gas vesicles were added in defined numbers to wild-type *Halobacterium salinarum *NRC-1 cells, and respective increases in OD_600_ were determined. We mimicked two points in the late stationary phase of Facciotti et al's. [12] experiment in which CFUs had decreased by making a sample of viable mid-log (OD_600_ = 0.5) cells at an OD_600_ that was equivalent to points in curve (Figure 1, Points A and B). To these samples, purified gas vesicles (resalted to 25% NaCl) were added in fixed amounts corresponding to 40, 80, and 120 GV released per cell. The total number of GVs added was determined by multiplying the number of GV released per cell by the number of cells that lysed in the stationary phase of Facciotti et al. [12] cultures. The OD_600_ of these purified-GV/cell mixtures were then measured. For points A and B, the addition of an equivalent of 40 GV per cell (2.45 and 2.70, respectively) most closely replicated the OD_600_ (2.51 and 2.48) observed in Facciotti et al. batch culture [12] (Figure 2) and are reasonably similar with the value of 54 GVs per cell released calculated earlier. Again, the value of 40 GV per cell is lower than what was predicted by Hechler and Pfeifer [1], most likely due to the fact that cells appear to begin lysing in early stationary phase when complete GV accumulation has not yet occurred.

Together, these data support two hypotheses proposed in Facciotti et al. [12]. The first is that free GV's released from *Halobacterium salinarum *NRC-1 are capable of scattering significant light at 600 nm. The second is that free GV alone, in quantities expected to be released from lysing cells in Facciotti et al. [12], can explain the increase in OD_600_ described in their manuscript [12].

### 3.2. Intracellular GVs Also Increase Light Scattering at 600 nm

**Figure 3:**
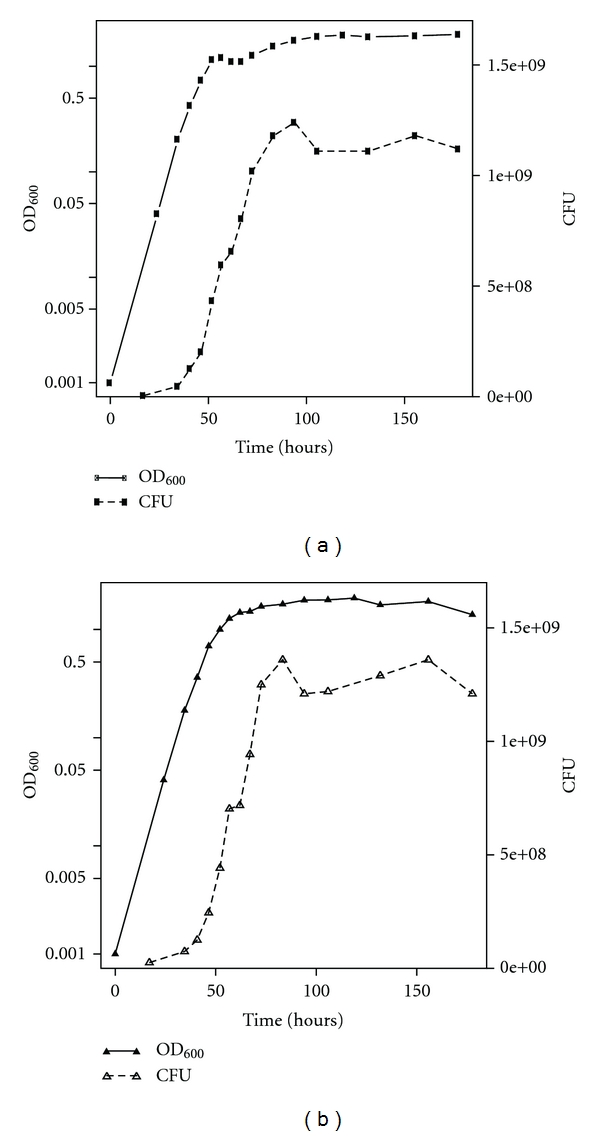
Growth of *Halobacterium salinarum *NRC-1 wild-type and GV deficient strains. *Halobacterium salinarum *NRC-1 wild-type and GV deficient strains were grown in triplicate as described in Materials and Methods. Growth was measured by light scattering at 600 nm (filled shapes) and by counting colony forming units using the spread plate technique (open shapes). OD_600_ is reported against the left *y*-axis in logarithmic scale and CFU is reported against the right *y*-axis in linear scale. (a) Growth of wild-type NRC-1 and (b) growth of GV deficient strain.

In addition to asking whether free gas vesicles could contribute to increases in OD_600_, we also sought to confirm whether or not intracellular gas vesicles in *Halobacterium salinarum* strains, as explained by Shand and Betlach [11] with unpublished data (a reference cited by others in this context), were indeed capable of explaining the stationary-phase-associated increase in OD_600_. We note that Walsby and coworkers [7, 17] have also shown light scattering by intracellular GV in *Halobacterium salinarum *strains. To quantify the influence of intracellular GV on scattering in *Halobacterium salinarum *NRC-1, we first isolated and grew a spontaneous and stable gas-vesicle minus (GV*−*) mutant of *Halobacterium salinarum NRC-1.* Wild-type *Halobacterium salinarum *NRC-1 and a mutant gas vesicle minus (GV*−*) strain were grown as described in Materials and Methods. Wild-type *Halobacterium salinarum *NRC-1 showed the stationary-phase-related increase in OD_600_ previously described in both Facciotti et al. and Shand and Betlach [11, 12] (Figure 3, Panel A). By contrast, the GV*−* strain lacked the stationary phase-associated increase in OD_600_ noted for wild-type samples (Figure 3, Panel B). The maximum CFU counts obtained from both wild-type and GV*−* cultures were ~1.5 × 10^9^ each. These data are consistent with the hypothesis that gas vesicle expression accounts for the increase in OD_600_ observed in the stationary phase of *Halobacterium salinarum *NRC-1.

**Figure 4:**
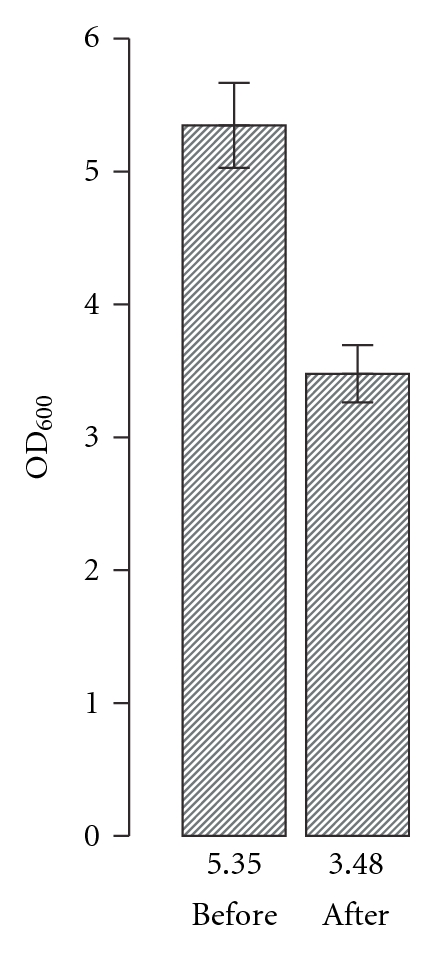
Light scattering by intracellular gas vesicles. Vacuolated cells were centrifuged at 5000 xg for 5 mins to deflate gas vesicles. OD_600_ and CFU counts were taken before and after centrifugation. No decrease in CFU or change in morphology were observed before and after centrifugation meaning that difference in OD_600_ can be directly attributed to gas vesicle loss. Error bars represent +/− one standard deviation of the quadruplicate measurements. OD_600_ values are included below each column as reference.

Second, we took vacuolated cells and “popped” the gas vesicles through centrifugation. In this case, cells that had an average initial OD_600_ = 5.35 were spun in a Eppendorf 5424 microcentrifuge (Eppendorf, Hamburg, Germany) at 5000 xg, for 5 minutes. Cells were then resuspended by gentle pipetting, and OD_600_ was measured to equal an average of 3.48. No decrease in CFUs determined before and after centrifugation was noted, ensuring viability had not changed. This suggests that the decrease in OD_600_ can be attributed to a depletion of gas vesicles and not cell lysis.  Figure 4 summarizes the data. Differential interference contrast images of cells taken before and after centrifugation show that, before centrifugation, nearly 95% of cells are highly vacuolated. The postcentrifugation images suggest that overall loss of GV is 50%. Given ~80 GV per cell, as reported by [1] for late stationary phase cells, 50% loss due to centrifugation, and no loss of viable cells due to centrifugation, we calculated that 2.32 × 10^10^ GVs were “popped” during centrifugation. Coupling this loss to the decrease in OD_600_ of 1.87 suggests that the GVSF for intracellular GV is 1.24 × 10^10^, a value 2.63 × 10^10^ less than the GVSF for free GVs. Alternatively, our calculations suggest that in *Halobacterium salinarum *NRC-1 free gas vesicles scatter 3.1X more when free than when they form intracellular gas vacuoles. An analogous difference in scattering magnitude between free and intracellular gas vesicles in *Anabaena flos-aquae *was reported to be 2.44X, respectively [25]. 

### 3.3. Sensitivity of Growth Curve Profiles on Culture Agitation and Geometry

During the course of testing the hypotheses above, we made two observations that seemed inconsistent with previously published data. First, *Halobacterium salinarum *NRC-1 cells grown in conditions that were intended to mimic culture conditions of Facciotti et al. [12] failed to lead to the same decrease in CFUs, despite reproducing an increase of similar scale in OD_600_ during stationary phase. Second, it was noted that the stationary phase-associated increase of OD_600_ reported by Shand and Betlach [11] for *Halobacterium salinarum *NRC817 was far greater than either of the experiments conducted herein or those reported by Facciotti et al. [12]. We sought to investigate these two issues.

 We hypothesized that the major differences (both in OD_600_ and CFUs) between our study and the aforementioned published studies could be at least partially explained by specific differences in growth conditions. This is particularly relevant when comparing our results to those of Facciotti et al. [12]. Comparing our data with that of Shand and Betlach [11] carries with it the additional complication that the two studies are growing similar but different strains of* Halobacterium salinarum*. Despite this key difference, it is nevertheless interesting to explore how much of the differences in final OD_600_ reported in each study could be accounted for in changing the growth conditions of flask-grown *Halobacterium salinarum *NRC-1. For instance, Shand and Betlach [11] reported that their cells were first grown in Erlenmeyer flasks at 350 rpm and subsequently shifted upon entry into stationary phase to tightly sealed Monogro flasks (Wheaton, NJ, USA) that were then shaken at 100 rpm in either highly illuminated or dark conditions. Facciotti et al. [12] grew their cultures in Erlenmeyer flasks in ambient light at 225 rpm on an Innova 4900 incubator shaker (New Brunswick, NJ, USA). Could, for instance, something as simple as reducing the shaking speed influence final OD_600_ in flask-grown *Halobacterium salinarum *NRC-1, lead to significantly higher final OD_600_?

**Figure 5:**
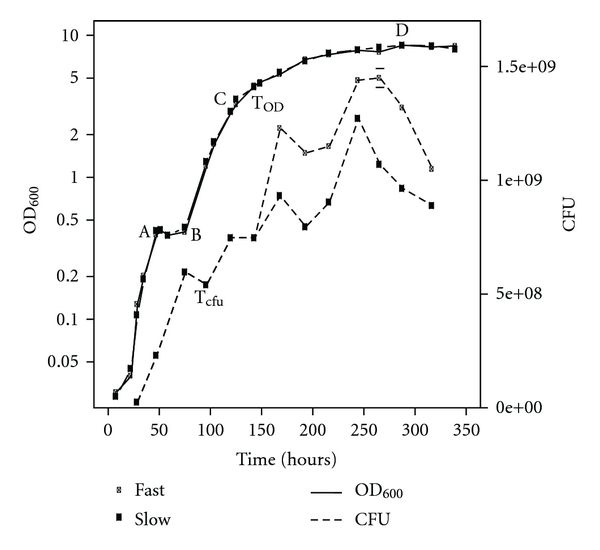
Mid-culture response of *Halobacterium salinarum *NRC-1 to changes in shaking speed. *Halobacterium salinarum *NRC-1 was grown at 100 rpm as described in Materials and Methods. Growth was tracked using both light scattering at 600 nm (solid lines) and by counting colony forming units using spread plate technique (dashed lines). OD_600_ is reported against the left *y*-axis in logarithmic scale and CFU is reported against the right *y*-axis in linear scale. Half of the sample set were transferred to 150 rpm at ~150 h. 100 rpm shaken cultures are shown as open circles and 150 rpm shaken cultures are shown as closed circles. Points A, B, C, and D refer to panels in Figure 6 in which the phenotype of the cultures are shown for the selected points. Error bars for CFU counts are included as +/− one standard deviation of 24 of 34 triplicate cultures. Error for time points without error bars were too small to display. Points marked with T indicated the points at which cultures were transferred from the slow shaking incubator to the fast shaking incubator.

**Figure 6:**
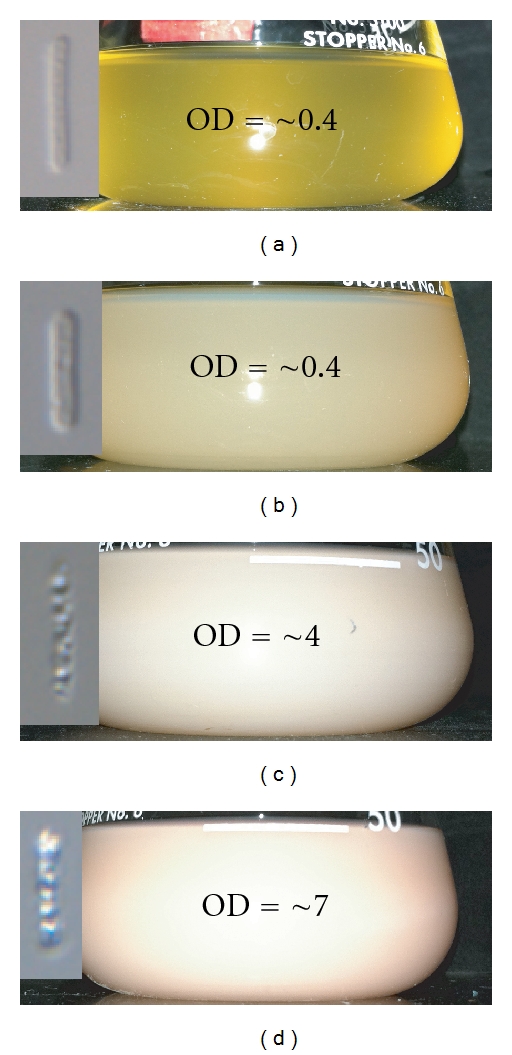
Cell and culture phenotypes under slow shaking conditions. *Halobacterium salinarum *NRC-1 cells were grown at 100 rpm as described in Materials and Methods. Phenotypes were tracked in slow shaken growth conditions by the culture vessel and by visible light microscopy of individual cells. Cell images are representative of >95% of the cultures' cell morphology at a specific time point. Time points were taken from Figure 5. (a) Culture and cell images from before the growth plateau, OD_600_ = ~0.4. (b) Culture and cell images from after the growth plateau, OD_600_ = ~0.4. (c) Culture and cell images from mid-stationary phase, OD_600_ = ~4.0. (d) Culture and cell images from late stationary phase, OD_600_ = ~7.0.

To test the hypothesis that a slower shaking speed can result in the divergent growth profile found by Shand and Betlach [11], where OD_600_ during stationary phase increased by ~9.0 units, we grew wild-type *Halobacterium salinarum *NRC-1 cells at 100 rpm simulating the agitation speed, though not the culture vessel, of the growth conditions in stationary phase reported by Shand and Betlach [11] (Figure 5). Under these growth conditions, we too saw a maximum OD_600_ of ~8.5 in stationary phase that approached the OD_600_ found by Shand and Betlach [11]. However, since we grew our culture at low rpm throughout the experiment (versus high speed until stationary), this growth curve was still different in two ways from what Shand and Betlach [11] reported. The first difference is that a short pause or transitory plateau in growth lasting ~24 hours occurs at OD_600_ ~0.4 in the flask culture shaken at 100 rpm on the Barnstead/LabLine Max^Q^ Mini 4450 (Thermo Scientific, Mass, USA). No evidence of a similar low-OD_600_ plateau is noted in any other growth experiment discussed in this manuscript so far. During this transition, the culture changes its appearance, becoming milky/pink at the end of the transition, a phenotypic change that is typically associated with GV accumulation [16]. Indeed while changes in the culture's visible appearance occur during this transition, microscopic analysis of cells before and after show gas vesicle accumulation occurs only after the transition (Figure 6).

 The accumulation of GVs in this case, however, seems to precede a second growth phase rather than just increasing scattering due to GV production and/or release. In addition to the appearance of transitory plateau in the growth curve of *Halobacterium salinarum *NRC-1, CFU counts increased beyond the plateau leading to the suggestion that the growth transition that occurs at OD_600_ = 0.4 can likely be attributed to physiological shifts in substrate utilization, a classic diauxic shift rather than not just the beginnings of GV accumulation. Since the only difference between the 100 and 225 rpm cultures (Figure 3, Panel A and Figure 5) was the shaking speed, we suspect that the diauxic transition may be influenced by the depletion of oxygen in the media. We note that oxygen solubility is extremely low in hyper-saline media. Beard et al. have reported the depth-dependent soluble oxygen concentration in static 4 M NaCl media, which has only a slightly lower NaCl concentration than that of CM (4.27 M NaCl). At this high NaCl concentration, dissolved oxygen at the surface is 66% that of ambient air (~45 *μ*mol/L) but falls precipitously to just 7.3% (~5 *μ*mol/L) in the first millimeter and ultimately to 3.1% (~2 *μ*mol/L) over the following 34 mm of depth [7]. Under such circumstances, it is reasonable to assume that the rate of oxygen consumption by the culture might quickly exceed the addition of new gas at slow shaking speeds. 

To test the hypothesis that oxygen depletion at low shaking speed induced the diauxic shift in *Halobacterium salinarum *NRC-1, we recultured wild-type *Halobacterium salinarum *NRC-1 cells at 100 rpm. However, during this culture, we transferred half of the cultures to a New Brunswick Scientific G-53 Shaker (New Brunswick Scientific, NJ, USA) at 150 rpm 75 hours after the presumed diauxic shift (Figure 5, Point T), assuming that this would increase the dissolved oxygen. The major impact of this instrument transfer is to increase aeration. Up until culture separation at 150 h, OD_600_ and CFU were completely identical for slow (100 rpm) and fast (150 rpm) shaken cultures. Seven hours after three flasks were placed at 150 rpm, CFU of the fast cultures was 1.25 × 10^9^ (viable cells/mL) while that of the slow cultures was 9.0 × 10^8^ CFUs (viable cells/mL), a difference of 3.5 × 10^8^ CFUs (viable cells/mL) (Figure 5). The quick transition to increased growth noted in the fast shaken cultures is consistent with the observations of Schmid et al. who noticed rapid resumption of growth in response to a sharp increase in dissolved oxygen [10]. Interestingly, while CFUs increased after the transition point in the fast shaken cultures with respect to their slow counterparts, we did not observe a significant change in OD_600_ between fast and slowly shaken cultures.

These data suggest that 100 rpm shaken cultures can become oxygen starved at OD_600_ nearing 0.4 and that the addition of dissolved oxygen introduced by transition to higher RPM shaking allows for a resumption in oxygen-dependent metabolism from substrates that are still present but would have otherwise been consumed had the culture been at high RPM the whole time. In addition, the indistinguishable values of OD_600_ between fast and slow shaken cultures suggest that the resumption of growth may have reduced per cell GV accumulation in the fast shaken samples.

The second reported discrepancy between results by Facciotti et al. [12] and our initial growth experiments in which cells appeared to lyse and remain intact, respectively, during stationary phase were more difficult to test directly. Here we hypothesize that small differences in media or culturing may play a role. In our initial experiments, while designed to mimic Facciotti et al. [12] cultures were shaken on a Barnstead/LabLine *Max*⁡^Q^ Mini 4450 (Thermo Scientific, Mass, USA) at 225 rpm instead of the Innova 4900. The different shaking platforms may have introduced some variation due to differences in shaking orbits which were 2 in and 0.75 in, respectively for the Innova 4900 and the Barnstead/LabLine Max^Q^ Mini 4450. It is possible that the additional agitation on the Innova 4900 induced cell lysis whereas growth on the LabLine Max^Q^ Mini 4450 did not though violently shaking 20 mL aliquots of our cells in 50 mL conical tubes did not lead to reduced cell viability. Media composition, including elements present in the local DI water sources may have also contributed to differences between our experiments and those of Facciotti et al. [12]. Finally, *Halobacterium *species are known to be sensitive to residual dishwashing detergent left on glassware. While wash protocols for samples collected by Facciotti et al. [12] and our current data both involve significant amounts of rinsing with DI water prior to use, it is possible that differences in detergent type or precise amount of rinsing may also contribute to the discrepancy we note. Unfortunately, this specific mystery remains unresolved.

### 3.4. Sensitivity of Growth Curve Profiles on Trace Element Concentration

Given the data presented up to this point, it is tempting to functionally associate an apparent growth plateau, either early or late in the growth curve of *Halobacterium salinarum *NRC-1, directly with gas vesicle production and to some extent with oxygen availability. However, we have, for some time, suspected that some of the small interexperiment differences we observe in the qualitative appearance of the growth plateau in *Halobacterium salinarum *NRC-1 might be related to the concentration of the trace elements Fe^2+^ and Mn^2+^ (unpublished data) rather than a direct effect of oxygen or gas vesicle accumulation. We, therefore, decided to formally test the influence of the typical trace elements Fe^2+^ and Mn^2+^ on the shape of the growth curve.

**Figure 7:**
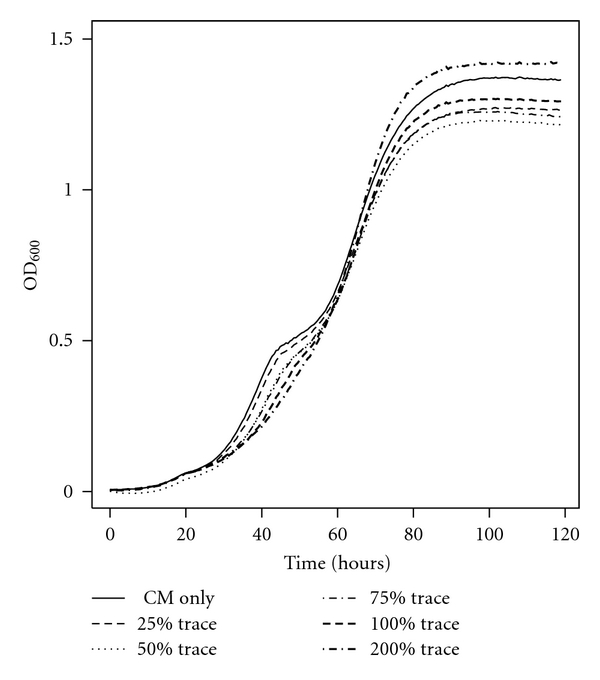
Influence of trace element concentration on the growth curve of *Halobacterium salinarum *NRC-1. *Halobacterium salinarum *NRC-1 was grown in CM with 0X, 0.25X, 0.5X, 0.75X, 1.0X and 2.0X standard Fe^2+^ and Mn^2+^ concentrations (18 *μ*M and 1.9 *μ*M) in a 96-well plate as described in Materials and Methods. Growth was tracked by OD_600_ and reported against the y-axis in linear scale to highlight phenotypic differences. OD_600_ values were converted to a path length of 1 cm to be equivalent with other optical density measurements in this manuscript. Each curve is an average of six replicates.

Since the most common growth media formulation used for *Halobacterium salinarum *NRC-1 calls for Fe^2+^ and Mn^2+^ to be added at 18 *μ*M and 1.9 *μ*M, respectively, we decided to test the influence of different dilutions of Fe^2+^ and Mn2+ stock solutions. 0.25X, 0.5X, 0.75X, 1.0X, and 2.0X equivalent amounts of Fe^2+^ and Mn^2+^ were added to CM. *Halobacterium salinarum *NRC-1 was inoculated into each media variant and loaded into a 96-well plate as described in Materials and Methods. We note that oxygen levels in 96-well plates tend to be lower than those of well-shaken flask cultures. Therefore, growth curves grown in 96-well plates are more environmentally related to those of slowly agitated flask-grown culture (e.g., the 100 RPM experiment noted earlier) than the typical fast-shaken culture. *Halobacterium salinarum *NRC-1 grown in CM with no added trace elements exhibited a short plateau in growth at an OD_600_ of ~0.5 which may be analogous to the plateau reported in Figures 1 and 4. Increasing trace element concentration reduced the duration of the plateau until it was completely nonexistent in the 2.0X concentration trace element growth curve. The data are summarized in Figure 7. Despite this change in growth profile, Fe^2+^ and Mn^2+^ concentrations appear to have no affect on maximum OD_600_ values muddling the direct affect of Fe^2+^, and Mn^2+^ may have been on the production of GVs. After removal from the plate reader, cells grown in each of the trace element concentrations displayed the floating GV+ phenotype, and no apparent difference in GV abundance can be perceived by phase contrast microscopy. These results, together with prior data, seem to suggest that the plateau we see in growth of *Halobacterium salinarum *NRC-1 is likely due to a combination of factors that include (Fe^2+^), (Mn^2+^), and/or (O_2_).

This data complicates the simple view of a simple functional linkage between oxygen concentration, the growth plateau, and GV production. Rather it seems that oxygen, trace elements, and metabolic status (as sensed perhaps by the presence or absence of key metabolites) all integrate somehow to regulate gas vesicle production. To summarize, (a) our slow shaking experiment demonstrates that oxygen is likely one limiting factor involved in the formation of a growth plateau in *Halobacterium salinarum *NRC-1; (b) increased oxygen (fast shaking) can minimize the plateau or shift it later in growth; (c) iron and/or manganese can also eliminate the plateau in low oxygen conditions; (d) when the plateau occurs, it is typically associated with increased GV production but its absence does not preclude GV production; (e) arginine and citrate can both influence GV production [1]. What is left to understand is whether or not all of these factors (oxygen, trace elements, and key metabolites) that appear to be either directly or indirectly linked to the regulation of gas vesicle biogenesis are somehow functionally coordinating GV expression. A more complete synthesis of some potential regulatory schemes that are consistent with these observations is presented in the discussion.

## 4. Discussion

In this manuscript, we started by addressing relatively simple questions regarding the ability of gas vesicles in *Halobacterium salinarum *NRC-1 to influence light scattering both intracellularly and free in solution. We have demonstrated that free GVs, in numbers that could be reasonably expected from lysing cells in batch grown culture, can scatter light sufficiently at 600 nm to account for the increase in OD_600_ that was previously noted to occur in stationary phase of *Halobacterium salinarum *NRC-1 by Facciotti et al. [12]. This conclusion now provides firm experimental support for the interpretation of the data reported in Facciotti et al. [12]. In addition, we provide direct evidence that intracellular gas vesicles also contribute significantly to increase in light scattering at 600 nm, a fact often proposed but for which little published evidence exists for *Halobacterium salinarum *NRC-1 [17, 25]. We also reaffirm the idea that careful characterization and reporting of culture conditions (including culture geometry and phenotype) is critical if we are to compare experimental data generated in different laboratories for gas vesicle production in *Halobacterium salinarum *NRC-1. This is highlighted by the fact that despite efforts to mimic growth conditions in Facciotti et al., cultures grown in our current laboratory setting did not show the decrease in CFUs noted by Facciotti et al. [12] in stationary phase. Additionally, imperceptible differences in OD_600_ between slow and fast shaken cultures (despite clear differences in growth) together suggest that CFU counts are particularly important to measure in experiments conducted with vacuolated cells.

More interestingly, these experiments have led us to think more about how specific environmental factors (some of which can be modulated by small changes in culture condition) influence growth phenotype and GV production. The data presented herein leads to the conclusion that while oxygen plays a crucial role in gas vesicle biogenesis in *Halobacterium salinarum *NRC-1 that other factors specifically trace elements and metabolites citrate and arginine (the latter results from Hechler and Pfeifer [1]) also play currently undefined but crucial roles. 

### 4.1. The Role of TbpD in the Regulation of Gas Vesicle Expression in *Halobacterium salinarum* NRC-1

The idea that factors other than oxygen likely play a role in the regulation of GV biosynthesis motivated us to review previously published data to determine whether or not we could begin to synthesize a regulatory scenario consistent with oxygen's role in GV biosynthesis, the observations made by Hechler and Pfeifer [1] regarding the influence of citrate, isocitrate, and arginine in the anaerobic production of GVs and the potential involvement of Fe^2+^ and/or Mn^2+^. At the very least, could we identify a limited set of potential “culprits” that could be associated with the regulation of GV biosynthesis? 

We were initially drawn to an interesting association between a gas vesicle biogenesis and a transitory growth plateau observed reported by Facciotti et al. [12] in a strain of *Halobacterium salinarum *NRC-1 in which the general transcription factor TbpD was constitutively expressed at nonnative levels, the *tbpD*-*cmyc *strain. The *tbpD-cmyc *strain, even when shaken at 225 rpm, grows more slowly than wild type and shows a similar, but shorter, transitory plateau in growth at OD_600_ ~0.4, as that seen in wild type shaken at 100 rpm (data presented in this manuscript). Moreover, GV accumulation is severely attenuated in the *tbpD-cmyc *strain relative to wild type. Microarray data also show that transcript abundance, particularly of genes transcribed from the pF promoter [23, 30], *gvpF,G,H,I,J,K,L, *and *M (vng5019g-vng5027g) *of *gvp*1 is also severely decreased in the *tbpD-cmyc *strain relative to wild type. Facciotti et al. [12] also report changes in GV transcript abundance in a *tbpD *knockout. Here, transcripts regulated by the pA and pD promoters, *gvpD,E,A,C,N, *and *O (vng5028g-vng5034g)* show decreased abundance relative to control strain levels, while transcripts abundance for genes regulated by the pF promoter, *gvpF,G,H,I,J,K,L, *and *M (vng5019g-vng5027g), *increased. Coker et al. [31] also report a decrease in expression of *gvpD,E,A,C,N, *and *O (vng5028g-vng5034g) *in the *tbpD*-knockout strains. We note that prior work also shows differential regulation of the pF and pD promoters. Northern blot analyses of the *gvp1 *gene cluster in *Halobacterium salinarum *sp. PHH1 have shown that genes transcribed from the pF promoter (*gvpFGHIJKLM*) are preferentially transcribed during exponential phase, while transcript from the pD promoter (*gvpDE*) are preferentially transcribed during stationary phase [32]. Together these data suggest a strong regulatory link between the general transcription factor TbpD and GV expression of the *gvp1 *gene cluster. 

Data from Facciotti et al. [12] for *tbpD *perturbation strains show little perceptible change in *gvp2 *(vng6229g-vng6246g) transcript abundance versus control strains, while data from Coker et al. suggest some perturbation of *gvp2 *genes in the *tbpD*-knockout strain [31]. Teufel et al. [33] have also shown that expression of the c-vac (*gvp2*) gene cluster is unperturbed in *Halobacterium salinarum *strain PHH4 (a strain lacking *tbps A, B C, D, and F).*

The slown growth and early diauxic shift seen in the *tbpD-cmyc *strain, which mimicked the diauxic shift seen in presumably oxygen-starved wild-type cells grown at 100 rpm in this study further suggested that *tbpD *expression may also be related to oxygen levels. We therefore looked for evidence of a link between perturbation in oxygen levels and *tbpD *expression, in particular for evidence that *tbpD *expression could be related to “low” oxygen conditions. Microarray data collected by Schmid et al. shows that *tbpD *transcript abundance increases under conditions where oxygen becomes limiting and that its abundance can be correspondingly decreased by shifting the cells from “low” to “high” oxygen concentrations [10]. Microarray data also shows that *tbpD *transcript abundance increases beginning in the stationary phase [12] when oxygen availability is thought to be limited. Data from Kaur et al. [14] further show that hydrogen peroxide stress also induces an increase in both *tbpD *and gas vesicle transcript abundance relative to unstimulated control samples. Kaur et al. propose in their manuscript that damaging levels of reactive oxygen species (ROS) trigger the cells to adopt a metabolic state similar to that adopted in oxygen depleted environments to minimize any additional damage induced by metabolically generated ROS. Given this interpretation, an increase in *tbpD *transcript abundance would be in agreement with the oxygen-dependent expression noted previously in Facciotti et al. [12] and in the data presented by Schmid et al. [10]. Together each of these observations suggests a functional link between TbpD, oxygen concentration, and regulation of GV biosynthesis.

### 4.2. The Role of Aconitase in the Regulation of Gas Vesicle Expression in *Halobacterium salinarum* NRC-1

We next integrate observations made in multiple manuscripts to show how the citric acid cycle enzyme aconitase, which catalyzes the reversible isomerization between citrate and isocitrate and in some organisms acts as a nucleic acid-binding protein [34–38], may also play an important role in the accumulation of gas vesicles. First, Facciotti et al. [12] noted that the transcript abundance of both aconitase and gas vesicle genes is low in the *tbpD-cmyc *strain compared to their levels in control strains. They also noted that in control strains, aconitase and *tbpD *transcript abundances are anticorrelated as cells transition through regions in the growth curve (i.e., lag, exponential growth and stationary phase). Cells entering the oxygen-depleted stationary phase down-regulate the expression of *aconitase *and concomitantly increase the expression of *tbpD*. In addition, the data from Schmid et al. [10] further support the observation that fluctuations in oxygen concentration can lead to anti-correlated transcriptional expression of aconitase and *tbpD*. Taken together, the data linking between *tbpD *expression and GV production, noted in the previous paragraph, the consistent anti-correlation between *tbpD *and aconitase in control cultures, and the perturbation of *aconitase *and GV expression in the *tbpD-cmyc *strain all appear to also link aconitase to GV production in *Halobacterium salinarum *NRC-1.

Interestingly, the links between the perturbation of GV phenotype, TbpD, and now to aconitase bring us back to the observations made by Hechler and Pfeifer [1] who observed that GV accumulation during anaerobic growth is also dependent on the presence either citrate or isocitrate, the respective substrate and product of aconitase. This preliminary functional inference for aconitase can also be interpreted in the context of other data. For instance, Kaur et al. [14] have observed a loss of gas vesicles in H_2_O_2_-induced stress in a peroxidase-knockout mutant with no concomitant change in GV transcript abundance. The increase in ROS species in the peroxidase-knockout mutant are thus likely acting to modulate GV abundance posttranscriptionally. This additional observation and the well-known sensitivity of aconitase to ROS, its known sensitivity to redox status in the cell [39], and its known role as an mRNA-binding regulatory protein in other species [35–37] also give additional credence to the hypothesis that aconitase may be involved in the regulation of GV biogenesis, perhaps in its capacity as an RNA-binding protein.

We also propose the hypothesis that the links between trace elements (Fe^2+^ and Mn^2+^) and gas vesicle production noted in results may also represent a link to aconitase function. The TCA cycle functions of aconitase are known to require the coordination of iron into the active site of the enzyme. However, in other organisms, aconitase has also been shown switch between its function as a TCA cycle catalyst and an iron and oxygen-sensitive nucleic acid-binding protein [35–37, 40]. Iron and oxygen-rich conditions favor the TCA cycle function while a conversion to a nucleic acid-binding form is favored in iron and oxygen depleted conditions. Using the program SIRE [41], we have discovered a putative iron responsive element (IRE)—a hairpin structure bound by aconitase in it role as an RNA chaperone—just downstream of *gvpK2.*

Many individual bits of evidence point to aconitase as potentially an important additional player in the regulation of GV biogenesis, perhaps serving as an integrator of metabolic and environmental signals that actively participating in regulation through oxygen, metabolite, and iron-dependent RNA-binding. While this hypothesis is plausible given all of mounting circumstantial evidence of aconitase's role in GV biogenesis, confirmation clearly awaits further experiment and analysis.

### 4.3. Integrating both Aconitase and TbpD Activity into a Model for GV Regulation

Integrating the roles of aconitase and TbpD into the model for the regulation of GV biogenesis is still challenging given the available data. However, we propose a hypothesis, consistent with the data discussed above, that can stimulate the design of future experiments and ultimately refine our understanding of GV biogenesis. First, as with aconitase in other organisms, we posit that aconitase in *Halobacterium salinarum *NRC-1 can function as an RNA chaperon to posttranscriptionally regulate GV biogenesis through the binding of an IRE or IRE like element. While the specific target of aconitase is unknown, the binding to RNA is hypothesized to facilitate the translation of the target, perhaps even GV transcripts. As in other systems, the switch in aconitase activity in *Halobacterium salinarum *NRC-1 between TCA cycle enzyme, and RNA-binding protein is likely also mediated by environmental factors like iron and oxygen, in which the RNA-binding function is favored under low oxygen concentration, high ROS species concentration and/or iron limitation. If the *Halobacterium salinarum *NRC-1 aconitase has similar sensitivity to environmental factors, it would then be driven to RNA binding under low oxygen conditions. Under such conditions, it could serve as a chaperone for GV transcript translation, consistent with the hypothesis that microaerobic or anaerobic conditions seem to drive GV biosynthesis and that part of this process is modulated posttranscriptionally. If aconitase serves such a role and the expression, stability, or activity of aconitase can also be further modulated by the presence of citrate or isocitrate, this could also help explain the observations of Hechler and Pfeifer [1]. 

One simple hypothesis for the role of TbpD, consistent with the current data, is to propose that TbpD acts through protein-protein interactions to modulate the activity of a yet unspecified transcriptional regulator. Teufel and Pfeifer have recently presented *in vitro *data showing that TbpD can physically interact with the activator GvpE [42]. While this was presented as evidence that TbpD might be capable of interacting with GvpE at promoters to initiate transcription, an alternate interpretation would be to propose that TbpD competes with an alternate, activating Tbp for GvpE binding thus modulating the effective GvpE activity. Another potential target for TbpD could also be one or more of the potential activating Tbps in *Halobacterium salinarum *NRC-1. Facciotti et al. have presented co-IP data showing that TbpD may be highly promiscuous in its interactions with alternate Tbps in *Halobacterium salinarum *NRC-1, interacting with general transcription factors TbpA, TbpB, TbpE, TbpF, and TfbC [43]. Tbp dimer formation has already been demonstrated with eukaryotic Tbps [44–46] and is thought to be a mechanism for regulating the pool of available Tbp. A combination of the previous two schemes could also modulate the activity of GvpE through a mechanism in which TbpD interacts with hetero or homodimers of alternative activating Tbps. Of course, other regulators may also be targets of TbpD activity.

This type of functional activity could easily explain the observations of the TbpD nonnative expression and knockout perturbation experiments noted earlier. Overexpression of TbpD results in severe downregulation of aconitase and GV transcripts. This could be accomplished through functional attenuation of a transcriptional regulator through physical interaction. Likewise, the apparent overexpression of genes downstream of the pF promoter, *gvpF,G,H,I,J,K,L and M (vng5019g-vng5027g), *could be explained by the a lack of modulated activity of the regulatory target in the TbpD-knockout strain. Verifying this type of activity for TbpD would also help to shed more light on the functional roles for the multiplicity of Tbp proteins in some of the archaea.

## 5. Conclusion

Despite the fact that the environmental and regulatory inputs that control GV biogenesis are not yet completely elucidated, what is clearly emerging is the idea that the process is highly sensitive to small environmental perturbations that may be introduced by both the experimentalist, and in natural settings, by the environment. The regulation of GV biosynthesis is clearly multidimensional, involving environmental factors such as dissolved oxygen concentration, specific nutrient availability (i.e., citrate, isocitrate, arginine), specific transcriptional regulators (GvpE, GvpD, TbpD, and perhaps several tfIIB homologs) and perhaps even the activity of the dual function TCA-cycle enzyme/RNA-binding protein aconitase. While the specific roles of all of the regulatory influences, both direct and indirect, are not yet known, the cluster of possible candidates is becoming incrementally better defined. This suggests that reporting detailed descriptions of experimental setup is critical for interpreting data related to GV biogenesis, and that some measurements of nutrient availability and oxygen concentration should be either explicitly measured or strictly controlled in future experiments. We propose that, due to the multidimensional nature of GVs biogenesis and its regulation, this system may in fact provide a good experimental system in which to study biological principles of environmental and genetic signal integration that occur both transcriptionally and posttranscriptionally for the assembly of complex intracellular structures.
